# IL-9 Induces VEGF Secretion from Human Mast Cells and IL-9/IL-9 Receptor Genes Are Overexpressed in Atopic Dermatitis

**DOI:** 10.1371/journal.pone.0033271

**Published:** 2012-03-08

**Authors:** Nikolaos Sismanopoulos, Danae A. Delivanis, Konstantinos D. Alysandratos, Asimenia Angelidou, Magdalini Vasiadi, Anastasia Therianou, Theoharis C. Theoharides

**Affiliations:** 1 Laboratory of Molecular Immunopharmacology and Drug Discovery, Department of Molecular Physiology and Pharmacology, Tufts University School of Medicine, Boston, Massachusetts, United States of America; 2 Department of Biochemistry, Tufts University School of Medicine, Boston, Massachusetts, United States of America; 3 Department of Internal Medicine, Tufts University School of Medicine and Tufts Medical Center, Boston, Massachusetts, United States of America; 4 Allergy Clinical Research Center, Allergy Section, Attikon General Hospital, Athens University Medical School, Athens, Greece; 5 First Department of Dermatology, A. Sygros Hospital, Athens University Medical School, Athens, Greece; French National Centre for Scientific Research, France

## Abstract

Interleukin 9 (IL-9) has been implicated in mast cell-related inflammatory diseases, such as asthma, where vascular endothelial growth factor (VEGF) is involved. Here we report that IL-9 (10–20 ng/ml) induces gene expression and secretion of VEGF from human LAD2. IL-9 does not induce mast cell degranulation or the release of other mediators (IL-1, IL-8, or TNF). VEGF production in response to IL-9 involves STAT-3 activation. The effect is inhibited (about 80%) by the STAT-3 inhibitor, Stattic. Gene-expression of IL-9 and IL-9 receptor is significantly increased in lesional skin areas of atopic dermatitis (AD) patients as compared to normal control skin, while serum IL-9 is not different from controls. These results imply that functional interactions between IL-9 and mast cells leading to VEGF release contribute to the initiation/propagation of the pathogenesis of AD, a skin inflammatory disease.

## Introduction

IL-9 was first described in the late 1980s as a member of a growing number of cytokines that has pleiotropic functions in the immune system [1]. IL-9 was initially purified and characterized as a T cell and mast cell growth factor [2]. IL-9 production was first associated with the Th2 phenotype, and many of the preliminary functions of IL-9 were tested in models of Th2-associated immunity [3]. Th17 cells, which are defined by secretion of IL-17A and IL-17F, may also secrete IL-9 *in vitro* and *ex vivo* [4], [5]. Mast cells also produce IL-9 in response to LPS and IL-1[6], [7]. TGF-beta and IL-4, that are secreted by mast cells, are potent cytokines in promoting the generation of IL-9-secreting cells [3], [8]. One of the main functions of IL-9 is to promote mast cell growth and function [9]. IL-9, alone or in combination with stem cell factor or FcεRI, promotes the expression of mast cell proteases and pro-allergic cytokines in cultured mast cells [10]–[12].

IL-9 demonstrates pro-inflammatory activity in several mouse models of inflammation and appears to play a significant role in the pathogenesis of atopic diseases and asthma [13], [14]. Transgenic expression of IL-9 in the lung results in allergic inflammation while blockade of IL-9 decreases allergic inflammation, mastocytosis and airway remodeling [15], [16] as well as inflammation [16]. IL-9 and IL-9R expression is increased in lungs of asthmatic patients, but not healthy controls [13], [17]. IL-9 increases susceptibility to passive or active systemic anaphylaxis [18]. Deficiency in IL-9 or IL-9R attenuates intestinal anaphylaxis, while transgenic expression of IL-9 in the intestine results in local mastocytosis and increased susceptibility to intestinal anaphylaxis [19], [20]. Apparently, IL-9 promotes mast cell mediated intestinal permeability and plays a role in the development of food allergies [20]. Many patients with moderate atopic dermatitis (AD) were shown to have high levels of sensitization to foods [21].

IL-9 could be involved in the pathogenesis of inflammatory skin disorders, such as AD, characterized by chronic skin inflammation that also involves mast cells [22]. Here we show that IL-9 gene expression is increased in lesional AD skin and stimulates VEGF release from cultured mast cells.

## Materials and Methods

### Reagents

Human IL-9 was purchased from Sigma (St. Louis, MO). STAT3 inhibitor Stattic was purchased from Santa Cruz Biotechnology (Santa Cruz, CA).

### Culture of human mast cells

LAD2 mast cells (kindly supplied by Dr. A.S. Kirshenbaum, National Institutes of Health, Bethesda, MD), derived from a human mast cell leukemia [23], were cultured in StemPro-34 medium (Invitrogen, Carlsbad, CA) supplemented with 100 U/ml penicillin/streptomycin and 100 ng/ml recombinant human stem cell factor (rhSCF, Stemgen, kindly supplied by Swedidh Orphan Biovitrum AB, (Stockholm, Sweden). Cells were maintained at 37°C in a humidified incubator at 5% CO2.

### VEGF release assay

LAD2 cells (1×105 cells/250 µl) were distributed in 96-well microtiter assay plates in triplicate and stimulated in complete culture medium with the indicated concentrations of IL-9. VEGF was determined in cell-free supernatants with a commercial ELISA kit (R&D Systems, Minneapolis, MN) according to the manufacturer's directions. VEGF secretion data are expressed as pg/106 cells. For inhibition studies, inhibitors were added to the media 30 min prior to stimulation

### ELISA analysis of STAT3 phosphorylation

LAD2 cells were plated in 24-well plates (3*103 cells/well) in complete media. Cells were stimulated with IL-9 for the indicated time-points. Stimulation was terminated by the addition of ice-cold PBS. Cells were washed once with PBS and then lysed in cell lysis buffer (#9803s, Cell Signaling Danvers, MA) and sonicated briefly. Equal amounts of protein from the cell lysates were used. Phospho-STAT3 levels were determined in the cell lysates with a commercial Elisa kit (Cell Signaling) according to the manufacturer's directions.

### Patients and biopsies

Full depth (3 mm3) punch skin biopsies were collected from subjects (patients and controls) who had not received any medication for 15 days prior to the biopsy and were seen at the 2nd Department of Dermatology of the Attikon General Hospital, Athens University Medical School, Athens, Greece. The Medical Ethics Committee of Attikon Hospital Institution's Human Investigation Review Board (HIRB) approved this protocol. All participants gave their written informed consent according to the Declaration of Helsinki Principles. Patients were free from any other medical problems. All biopsies (patients and controls) were obtained from non-exposed skin (back and gluteal) and were immediately placed in RNAlater solution (Ambion, Inc., Austin, Texas, USA) and stored at −20°C.

### PCR and quantitative PCR

Total RNA from skin biopsies or cultured mast cells was isolated using Trizol (Invitrogen), according to the manufacturer's instructions. Reverse transcription was performed with 200 ng of total RNA using the iScript cDNA synthesis kit (Ambion, Austin, TX).

In order to measure IL-9, and IL-9 receptor (IL-9r) gene expression, quantitative real time PCR was performed using Taqman gene expression assays. The following probes obtained from Applied Biosystems, were used: IL-9, (ID: Hs00914237_m1); IL-9 receptor (ID: Hs01108522_m1); Human GAPD (GAPDH) Endogenous Control (VIC/TAMRA Probe, Primer Limited), (Number: 4310884E). The cycling conditions consisted of 1 cycle of 50°C for 2 min, 1 cycle of 95°C for 10 min, 40 cycles of 95°C for 15 s, 1 cycle of 60°C for 1 min, 1 cycle of 95°C for 15 s, 1 cycle of 60°C of 30 sec and 1 cycle of 95 for 15 s. Relative mRNA abundance was determined from standard curves run with each experiment, and IL-9, and IL-9 receptor expression was normalized to GAPDH endogenous control.

## Results

### IL-9 stimulates VEGF production in human mast cells

**Figure 1:**
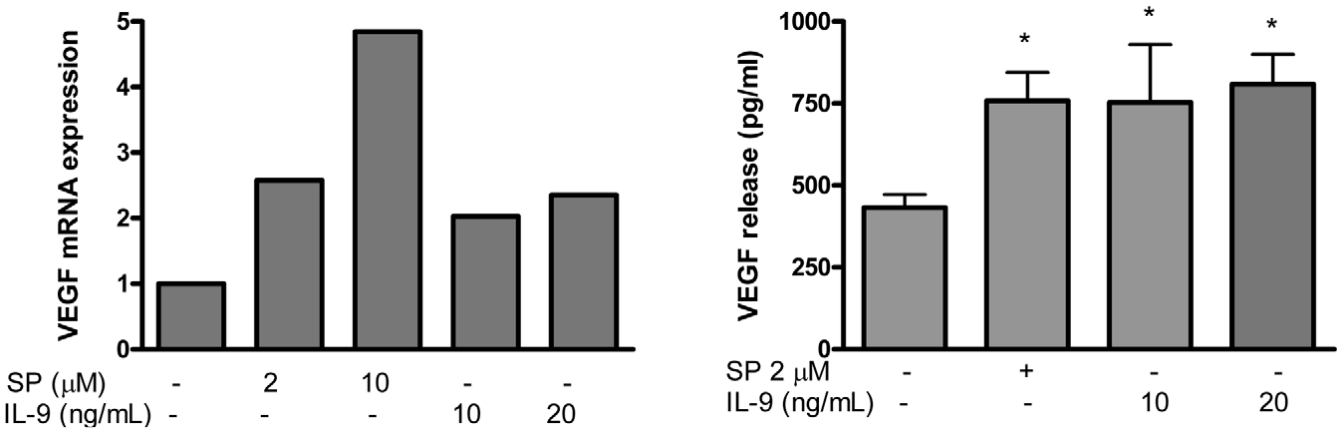
IL-9 stimulates VEGF production in human mast cells. (A) Gene expression. LAD2 cells were stimulated with IL-9 for 6 hrs, RNA was extracted and relative VEGF mRNA levels were determined by real-time PCR. (B) Protein release. LAD2 cells were stimulated with the indicated concentration of IL-9 (10–20 ng/ml) for 48 hrs. VEGF was measured in the supernatant fluid by ELISA. Data are the mean ± SD of 3 separate experiments performed in triplicate (*P<0.05 versus unstimulated cells).

To examine the effect of IL-9 on VEGF secretion, LAD2 cells were treated with IL-9 (10, 20 ng/ml) for 48 hr. VEGF mRNA (measured at 6 hrs) was increased after IL-9 stimulation (Fig. 1A). IL-9 also stimulated release of VEGF with a maximum of 860 pg/106 cells at 10 ng/ml, a 2-fold induction (Fig. 1B). There was no apparent difference between 10 and 20 ng/ml IL-9 and lower concentrations did not have any effect. There was no degranulation as measured by beta-hexosaminidase release or release of IL-1, IL-8 or TNF (results not shown).

### STAT3 Phosphorylation and activation is involved in IL-9-induced VEGF release

**Figure 2:**
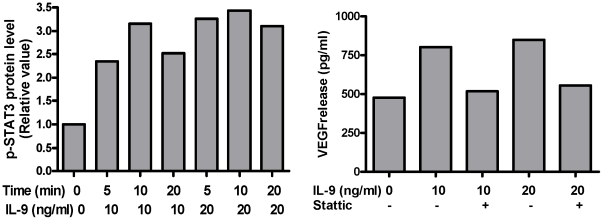
(A) IL-9 induces STAT3 phosphorylation in LAD2 cells. Cells were stimulated with IL-9 for up to 20 min. Phospho-STAT3 levels in the cell lysates were determined by ELISA. (B) STAT3 inhibitor Stattic inhibits IL-9-induced VEGF release from LAD2 cells. LAD2 cells were pre-incubated for 30 min with the indicated concentrations of Stattic. Cells were stimulated with IL-9 (10–20 ng/ml) for 24 h and supernatant VEGF was measured by ELISA. Data are representative of similar experiments.

We investigated downstream events associated with stimulus-receptor coupling. Stimulation with IL-9 (10 ng/ml) for 5,10, or 20 min, increased STAT3 phosphorylation, which was detected within 5 min (Fig. 2A). Pretreatment with the inhibitor of the STAT3 pathway, Stattic, blocked IL-9-induced VEGF release without significantly affecting basal release (Fig. 2B). Together, these data demonstrate that IL-9 induces phosphorylation of STAT3 in human mast cells and activation of STAT3 is necessary for VEGF production.

### IL-9 and IL-9 receptor mRNA expression is increased in skin of patients with AD

**Figure 3:**
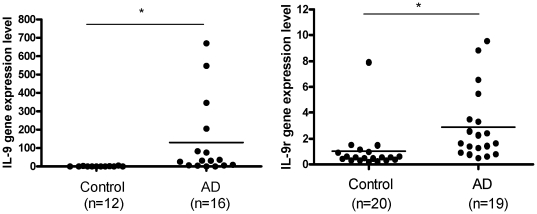
(A) IL-9 gene expression in the skin of AD affected (n = 16) and normal healthy controls (n = 12). (B) IL-9 receptor (IL-9r) gene expression in skin of AD affected (n = 19) skin and normal healthy controls (n = 20). Relative quantities of mRNA expression were measured by quantitative RT-PCR and normalized to GAPDH. TaqMan was performed with cDNA reverse transcribed from 100 ng RNA from each sample. *p<0.05.

Analysis of skin biopsies from AD lesional (n = 12) and control normal skin (n = 16) showed that IL-9 (Fig. 3A) IL-9r (Fig. 3B) gene expression is significantly higher in lesional AD skin than normal control skin.

### IL-9 levels in serum of patients with AD

**Figure 4:**
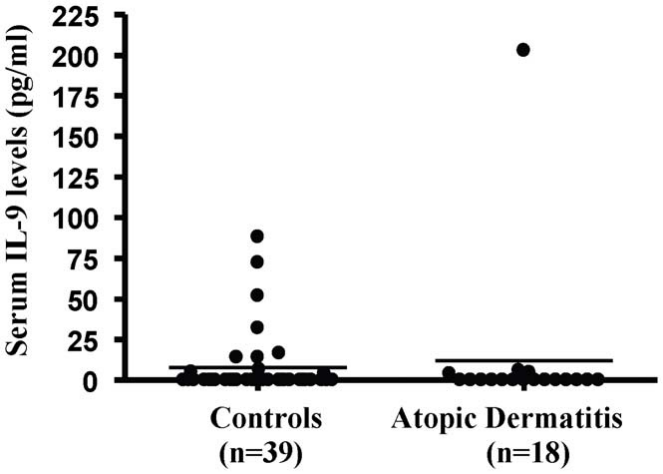
IL-9 serum level in AD patients (n = 18) and controls (n = 39). IL-9 level was determined using Millipex microbead assay and the measurements were performed blind by Millipore (St. Charles, MI).

Serum levels of IL-9 were not different in AD patients as compared to controls (Fig. 4).

## Discussion

We report here for the first time that IL-9 induces VEGF release from human mast cells. This effect is greater than what has been previously reported for IL-33 [24]. IL-9 cannot stimulate human mast cell degranulation on its own or the production of other cytokines like IL-1, TNF, or IL-8. The amount of IL-9 inducing maximal VEGF release was 10 ng/ml, while 0.1 and 1 ng/ml had no effect (results not shown). The concentration we used (10 ng/ml) may be far highr than the concentrations needed for responses in some IL-9 dependent cell lines, but less than the dose (20 ng/ml) used for differentiation of Th17 cells [5], and lower than the dose (30 ng/ml) shown to stimulate cytokin egene expression from rodent mast cells [25]. The combination of SP and IL-9 did not produce any highr release than either trigger alone (results not shown). Here we also show for the first time that gene expression of IL-9 and IL-9 receptor are increased in lesional AD skin. In contrast, Il-9 was not increased in AD patients. A recent paper investigating serum IL-9 levels in systemic sclerosis, where it was elevated, also reported no difference between AD levels and controls [26].

IL-9 is a cytokine produced especially by CD4+ T-cells, but it appears that mast cells are also capable of producing IL-9 [7]. IL-9r is mostly expressed on mast cells [27], and also by regulatory T (TReg) cells, T helper 17 (TH17) cells and antigen-presenting cells [28]. Even though IL-9 is mostly associated with mast cell growth [2], it also induces expression of pro-inflammatory cytokines [25], and release of IL-6 without degranulation and without mast cell proteases from mouse bone marrow-derived mast cells [29]. IL-9 could also have a synergistic effect with allergic and non-allergic triggers on activating mast cells and basophils [28]. IL-9 was recently shown to be involved in the development of food allergies, conditions known to also implicate mast cells [20].

We also show that the effect of IL-9 on mast cells VEGF release requires STAT3 activation. It is interesting that STAT6 was recently shown to be involved in both promotion of Th9 development through distinct transcription factors, activated by TGF-β and IL-4 [30]. The ability of IL-9 to induce mast cell release of VEGF is relevant to AD since it was demonstrated that the levels of dermal angiogenesis correlate with the inflammation observed in AD mouse models [31]. VEGF levels are also increased in AD compared to normal skin [32] especially the VEGF 121 isoform that causes vascular permeability [32], [33]. A recent paper reported that a number of IL-9 and IL-9 r gene polymorphisms in a Korean population are associated with increased risk of developing AD [34]. VEGF belongs to a family of heparin binding growth factors and is a major pro-angiogenic factor involved in many inflammatory diseases [35]. The progression of inflammation parallels the dermal angiogenesis in murine models of atopic dermatitis [31]. The VEGF 121 isoform also causes vascular permeability [36], [37]. Mast cells can secrete VEGF in response to IgE [38], substance P (SP) [24], and corticotropin-releasing hormone (CRH) [39], secreted under stress.

Mast cells are found in large numbers in around blood vessels in the skin where they participate in allergic and inflammatory reactions through release of multiple mediators with potent vasodilatory, inflammatory and nociceptive properties [40]. In addition to VEGF, histamine increases vascular permeability [41] and stimulates cutaneous sensory nerves [42] contributing to pruritus. In acute AD lesions, mast cells are normal in number but they appear to be degranulated [43]. In chronic lesions, however, especially in areas of lymphocytic infiltration in the papillary dermis, mast cells numbers are significantly increased, in close association with endothelial cells [44], [45]. Mast cell activation levels were shown to be correlated with the severity of AD [46]. Skin mast cells may have important functions as “sensors” of environmental and emotional stress [47].

The present results is the first indication that interactions between IL-9 and mast cells may be important in inflammatory skin diseases [48] where there is increased angiogenesis, such as AD. They may also represent novel therapeutic targets.

### Statistical analysis

Data are expressed as the mean ± SD. Statistical significance between experimental samples and controls was calculated using the Student's t-test. *P*<0.05 was considered statistically significant.

## References

[1] Hultner L, Druez C, Moeller J, Uyttenhove C, Schmitt E (1990). Mast cell growth-enhancing activity (MEA) is structurally related and functionally identical to the novel mouse T cell growth factor P40/TCGFIII (interleukin 9).. Eur J Immunol.

[2] Uyttenhove C, Simpson RJ, Van SJ (1988). Functional and structural characterization of P40, a mouse glycoprotein with T-cell growth factor activity.. Proc Natl Acad Sci U S A.

[3] Temann UA, Ray P, Flavell RA (2002). Pulmonary overexpression of IL-9 induces Th2 cytokine expression, leading to immune pathology.. J Clin Invest.

[4] Nowak EC, Weaver CT, Turner H, Begum-Haque S, Becher B (2009). IL-9 as a mediator of Th17-driven inflammatory disease.. J Exp Med.

[5] Elyaman W, Bradshaw EM, Uyttenhove C, Dardalhon V, Awasthi A (2009). IL-9 induces differentiation of TH17 cells and enhances function of FoxP3+ natural regulatory T cells.. Proc Natl Acad Sci U S A.

[6] Hültner L, Kölsch S, Stassen M, Kaspers U, Kremer J (2000). In activated mast cells, IL-1 up-regulates the production of several Th2-related cytokines including IL-9.. J Immunol.

[7] Stassen M, Muller C, Arnold M, Hultner L, Klein-Hessling S (2001). IL-9 and IL-13 production by activated mast cells is strongly enhanced in the presence of lipopolysaccharide: NF-kappa B is decisively involved in the expression of IL-9.. J Immunol.

[8] Schmitt E, Germann T, Goedert S, Hoehn P, Huels C (1994). IL-9 production of naive CD4+ T cells depends on IL-2, is synergistically enhanced by a combination of TGF-beta and IL-4, and is inhibited by IFN-gamma.. J Immunol.

[9] Townsend JM, Fallon GP, Matthews JD, Smith P, Jolin EH (2000). IL-9-deficient mice establish fundamental roles for IL-9 in pulmonary mastocytosis and goblet cell hyperplasia but not T cell development.. Immunity.

[10] Eklund KK, Ghildyal N, Austen KF, Stevens RL (1993). Induction by IL-9 and suppression by IL-3 and IL-4 of the levels of chromosome 14-derived transcripts that encode late-expressed mouse mast cell proteases.. J Immunol.

[11] Wiener Z, Falus A, Toth S (2004). IL-9 increases the expression of several cytokines in activated mast cells, while the IL-9-induced-IL-9-production is inhibited in mast cells of histamine-free transgenic mice.. Cytokine.

[12] Lora JM, Al-Garawi A, Pickard MD, Price KS, Bagga S (2003). FcepsilonRI-dependent gene expression in human mast cells is differentially controlled by T helper type 2 cytokines.. J Allergy Clin Immunol.

[13] Shimbara A, Christodoulopoulos P, Soussi-Gounni A, Olivenstein R, Nakamura Y (2000). IL-9 and its receptor in allergic and nonallergic lung disease: increased expression in asthma.. J Allergy Clin Immunol.

[14] Levitt RC, McLane MP, MacDonald D, Ferrante V, Weiss C (1999). IL-9 pathway in asthma: new therapeutic targets for allergic inflammatory disorders.. J Allergy Clin Immunol.

[15] Kearley J, Erjefalt JS, Andersson C, Benjamin E, Jones CP (2010). IL-9 Governs Allergen-induced Mast Cell Numbers in the Lung and Chronic Remodeling of the Airways.. Am J Respir Crit Care Med.

[16] Cheng G, Arima M, Honda K, Hirata H, Eda F (2002). Anti-interleukin-9 antibody treatment inhibits airway inflammation and hyperreactivity in mouse asthma model.. Am J Respir Crit Care Med.

[17] Bhathena PR, Comhair SA, Holroyd KJ, Erzurum SC (2000). Interleukin-9 receptor expression in asthmatic airways In vivo.. Lung.

[18] Knoops L, Louahed J, Van SJ, Renauld JC (2005). IL-9 promotes but is not necessary for systemic anaphylaxis.. J Immunol.

[19] Osterfeld H, Ahrens R, Strait R, Finkelman FD, Renauld JC (2010). Differential roles for the IL-9/IL-9 receptor alpha-chain pathway in systemic and oral antigen-induced anaphylaxis.. J Allergy Clin Immunol.

[20] Forbes EE, Groschwitz K, Abonia JP, Brandt EB, Cohen E (2008). IL-9- and mast cell-mediated intestinal permeability predisposes to oral antigen hypersensitivity.. J Exp Med.

[21] Werfel T, Breuer K (2004). Role of food allergy in atopic dermatitis.. Curr Opin Allergy Clin Immunol.

[22] Liu FT, Goodarzi H, Chen HY (2011). IgE, Mast Cells, and Eosinophils in Atopic Dermatitis.. Clin Rev Allergy Immunol.

[23] Kirshenbaum AS, Akin C, Wu Y, Rottem M, Goff JP (2003). Characterization of novel stem cell factor responsive human mast cell lines LAD 1 and 2 established from a patient with mast cell sarcoma/leukemia; activation following aggregation of FcepsilonRI or FcgammaRI.. Leuk Res.

[24] Theoharides TC, Zhang B, Kempuraj D, Tagen M, Vasiadi M (2010). IL-33 augments substance P-induced VEGF secretion from human mast cells and is increased in psoriatic skin.. Proc Natl Acad Sci U S A.

[25] Wiener Z, Falus A, Toth S (2004). IL-9 increases the expression of several cytokines in activated mast cells, while the IL-9-induced IL-9 production is inhibited in mast cells of histamine-free transgenic mice.. Cytokine.

[26] Yanaba K, Yoshizaki A, Asano Y, Kadono T, Sato S (2011). Serum interleukin 9 levels are increased in patients with systemic sclerosis: association with lower frequency and severity of pulmonary fibrosis.. J Rheumatol.

[27] Renauld J-C, Kermouni A, Vink A, Louahed J, Van Snick J (1995). Interleukin-9 and its receptor: Involvement in mast cell differentiation and T cell oncogenesis.. J Leukocyte Biol.

[28] Goswami R, Kaplan MH (2011). A brief history of IL-9.. J Immunol.

[29] Hultner L, Moeller J (1990). Mast cell growth-enhancing activity (MEA) stimulates interleukin 6 production in a mouse bone marrow-derived mast cell line and a malignant subline.. Exp Hematol.

[30] Goswami R, Jabeen R, Yagi R, Pham D, Zhu J (2012). STAT6-Dependent Regulation of Th9 Development.. J Immunol.

[31] Chen L, Marble DJ, Agha R, Peterson JD, Becker RP (2008). The progression of inflammation parallels the dermal angiogenesis in a keratin 14 IL-4-transgenic model of atopic dermatitis.. Microcirculation.

[32] Zhang Y, Matsuo H, Morita E (2006). Increased production of vascular endothelial growth factor in the lesions of atopic dermatitis.. Arch Dermatol Res.

[33] Zhang Y, Furumura M, Morita E (2008). Distinct signaling pathways confer different vascular responses to VEGF 121 and VEGF 165.. Growth Factors.

[34] Namkung JH, Lee JE, Kim E, Park GT, Yang HS (2011). An association between IL-9 and IL-9 receptor gene polymorphisms and atopic dermatitis in a Korean population.. J Dermatol Sci.

[35] Dvorak HF, Brown LF, Detmar M, Dvorak AM (1995). Vascular permeability factor/vascular endothelial growth factor, microvascular hyperpermeability, and angiogenesis.. Am J Pathol.

[36] Creamer D, Allen MH, Groves RW, Barker JN (1996). Circulating vascular permeability factor/vascular endothelial growth factor in erythroderma.. Lancet.

[37] Creamer D, Allen M, Jaggar R, Stevens R, Bicknell R (2002). Mediation of systemic vascular hyperpermeability in severe psoriasis by circulating vascular endothelial growth factor.. Arch Dermatol.

[38] Boesiger J, Tsai M, Maurer M, Yamaguchi M, Brown LF (1998). Mast cells can secrete vascular permeability factor/vascular endothelial cell growth factor and exhibit enhanced release after immunoglobulin E-dependent upregulation of Fcε receptor I expression.. J Exp Med.

[39] Cao J, Papadopoulou N, Kempuraj D, Boucher WS, Sugimoto K (2005). Human mast cells express corticotropin-releasing hormone (CRH) receptors and CRH leads to selective secretion of vascular endothelial growth factor.. J Immunol.

[40] Theoharides TC, Alysandratos KD, Angelidou A, Delivanis DA, Sismanopoulos N (2010). Mast cells and inflammation.. Biochim Biophys Acta.

[41] Theoharides TC, Singh LK, Boucher W, Pang X, Letourneau R (1998). Corticotropin-releasing hormone induces skin mast cell degranulation and increased vascular permeability, a possible explanation for its pro-inflammatory effects.. Endocrinology.

[42] Christian EP, Undem BJ, Weinreich D (1989). Endogenous histamine excites neurones in the guinea-pig superior cervical ganglion *in vitro*.. J Physiol.

[43] Kawakami T, Ando T, Kimura M, Wilson BS, Kawakami Y (2009). Mast cells in atopic dermatitis.. Curr Opin Immunol.

[44] Soter NA (1989). Morphology of atopic eczema.. Allergy.

[45] Irani AM, Sampson HA, Schwartz LB (1989). Mast cells in atopic dermatitis.. Allergy.

[46] Kim YI, Neher E (1988). IgG from patients with Lambert-Eaton Syndrome blocks voltage- dependent calcium channels.. Science.

[47] Geck P, Maffini MV, Szelei J, Sonnenschein C, Soto AM (2000). Androgen-induced proliferative quiescence in prostate cancer: the role of AS3 as its mediator.. Proc Natl Acad Sci USA.

[48] Metz M, Maurer M (2009). Innate immunity and allergy in the skin.. Curr Opin Immunol.

